# Cerebellar-limbic neurocircuit is the novel biosignature of physio-cognitive decline syndrome

**DOI:** 10.18632/aging.104135

**Published:** 2020-11-25

**Authors:** Li-Kuo Liu, Kun-Hsien Chou, Chih-Chin Heather Hsu, Li-Ning Peng, Wei-Ju Lee, Wei-Ta Chen, Ching-Po Lin, Chih-Ping Chung, Pei-Ning Wang, Liang-Kung Chen

**Affiliations:** 1 Aging and Health Research Center, National Yang Ming University, Taipei, Taiwan; 2 Center for Geriatrics and Gerontology, National Yang Ming University, Taipei, Taiwan; 3 Brain Research Center, National Yang Ming University, Taipei, Taiwan; 4 Institute of Neuroscience, National Yang Ming University, Taipei, Taiwan; 5 Department of Biomedical Imaging and Radiological Sciences, National Yang Ming University, Taipei, Taiwan; 6 Institute of Public Health, National Yang Ming University, Taipei, Taiwan; 7 Department of Family Medicine, Taipei Veterans General Hospital, Yuanshan Branch, Yilan, Taiwan; 8 Department of Neurology, School of Medicine, National Yang Ming University, Taipei, Taiwan; 9 Department of Neurology, Taipei Veterans General Hospital, Taipei, Taiwan

**Keywords:** frailty, cognitive impairment, magnetic resonance imaging, brain volume, diffusion-weighted tractography

## Abstract

Both physical and cognitive deficits occur in the aging process. We operationally defined the phenomenon as physio-cognitive decline syndrome (PCDS) and aimed to decipher its corresponding neuroanatomy patterns and neurocircuit. High resolution 3T brain magnetic resonance imaging (MRI) images from a community-dwelling longitudinal aging cohort were analysed. PCDS was defined as weakness (handgrip strength) and/or slowness (gait speed) concomitant with impairment in any cognitive domain (defined by 1.5 standard deviation below age, sex-matched norms), but without dementia or disability. Among 1196 eligible ≥ 50-year-old (62±9 years, 47.6%men) subjects, 15.9% had PCDS. Compared to the other participants, individuals with PCDS had significantly lower gray-matter volume (GMV) in the bilateral amygdala and thalamus, right hippocampus, right temporo-occipital cortex, and left cerebellum VI and V regions. The regions of reduced GMV in people with PCDS were similar between the middle-aged and older adults; whereas larger clusters with more extensive GMV-depleted regions were observed in ≥65-year-olds with PCDS. Diffusion-weighted tractography showed disrupted hippocampus-amygdala-cerebellum connections in subjects with PCDS. The neuroanatomic characteristics revealed by this study provide evidence for pathophysiological processes associated with concomitant physio-cognitive decline in the elderly. This neurocircuit might constitute a target for future preventive interventions.

## INTRODUCTION

Aging is manifested in multi-morbidity, functional declining and disability which bring social burden. The World Health Organization highlights approaches to healthy aging that promotes functional and intrinsic capacity by preventing both physical disability and dementia [1]. Although the development of physical disability and dementia share many common risk factors and characteristics, these conditions in regard to their pathophysiology, preventive or pharmacotherapeutic interventions have usually been researched separately. Given their commonalities, multidomain interventions to tackle declined physical and cognitive functions simultaneously have been developed in recent studies with positive preliminary results [2, 3].

Physical frailty, an indicator of physical vulnerability and adverse outcomes in older people [4], not only presages physical disability but has also been associated with cognitive impairment [5]. Physical frailty and cognitive impairment may synergistically conduce to adverse health outcomes [6]. The International Association for Gerontology and Geriatrics and International Academy of Nutrition and Aging have proposed the concept of “cognitive frailty” to highlight the adverse impact of possible-reciprocal declines in physical and cognitive function [7], operationally defining cognitive frailty as concurrent physical frailty and mild cognitive impairment (MCI). Due to low prevalence and ineffective identification of at-risk subjects [8–10], Ruan et al. modified the operational criteria, defining pre-frailty/frailty plus subjective cognitive decline as “reversible cognitive frailty” and pre-frailty/frailty plus MCI as “potentially reversible cognitive frailty” [10]. This refinement highlighted the consideration of reversibility; however, no specific intervention trials have yet supported the reversibility of these modified criteria for cognitive frailty. Meanwhile, Verghese et al. proposed “motoric cognitive risk syndrome” (MCRS), which is defined by slow gait plus subjective cognitive complaints [11–13]. The epidemiology of MCRS and its risk for dementia, as well as ability to predict the transition to dementia, have been reported [11–13]. However, six years after its first description, clinical or research applications of MCRS remain obscure due to its inconsistent definitions of subjective cognitive complaints among different studies and yet unclear pathophysiology [13].

Our serial studies from the I-Lan Longitudinal Aging Study (ILAS), a community-based cohort of people aged ≥ 50 years without physical disability or dementia, have provided clues to the pathophysiology of interactions between physical and cognitive declines in the aging process [14–17]. The characteristics of ILAS include an extensive, objective neuropsychological assessment with multiple cognitive domains. We are the first to provide evidences implying the existence of frailty subtypes with specific etiology [14]. Mobility frailty, defined as slowness plus weakness, but not non-mobility frailty, defined as fatigue plus involuntary weight loss, were associated with poorer cognitive performance in one or more cognitive domains [14]. In addition, people with mobility frailty had a higher likelihood of mortality in the 3-year follow-up [14]. Brain magnetic resonance imaging (MRI) studies have further shown that mobility frailty was strongly associated with cerebellar gray-matter volume (GMV) deficits in both ILAS and a Japanese cohort population [17, 18], signifying a specific underlying pathogenesis. Therefore, we modified the diagnostic criteria for cognitive frailty according to these observations from our previous studies. We defined the physical-deficit part as weakness and/or slow; and cognitive-deficit part as cognitive performance in any domain that is at least 1.5 SD below the mean for age-, sex-, and education-matched norms. Based on this revised criteria, the prevalence of cognitive frailty in community-dwelling older adults was approximately 10–15 %, and those affected indeed had significantly higher risks of all-cause and cardiovascular mortality, and incident dementia [9, 15, 19, 20]. In terms of reversibility, sub-group analysis of a community-based intervention cohort in a cluster-randomized trial affirmed the efficacy of a multidomain intervention in preventing physio-cognitive decline in older adults [21]. Thus, this operational definition, which could identify a substantial proportion of at-risk people in the community, may have a unique patho-etiology and is potentially reversible. We have coined this modified cognitive frailty as “physio-cognitive decline syndrome” (PCDS) and formally proposed at the 5^th^ Asian Conference of Frailty and Sarcopenia in 2019 (https://www.agingmedhealthc.com/wp-content/uploads/2019/10/ACFS2019_Abstract-Book.pdf) [22].

The present study analysed brain MRI data from the ILAS population, aimed to investigate the potential neuroanatomic and neurocircuit correlates of PCDS. The results would provide neurobiological basis of PCDS and shed lights on the pathophysiology of the interactions between physical and cognitive declines in the aging process.

## RESULTS

### Demographic characteristics

**Figure 1:**
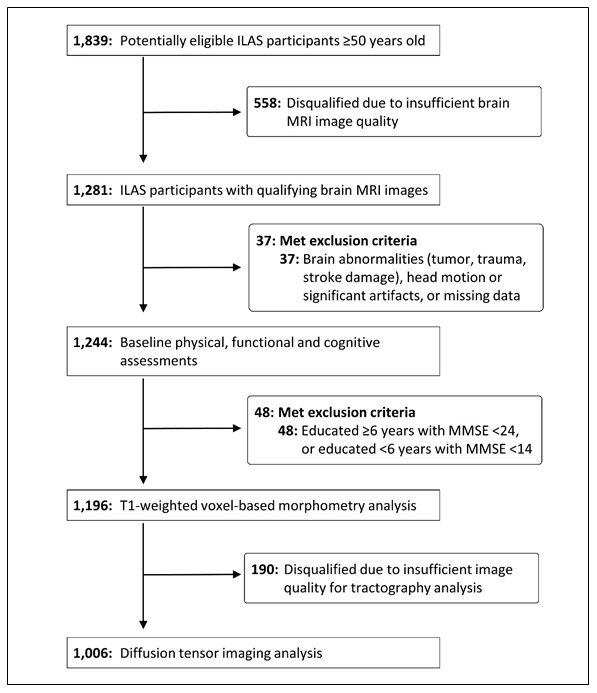
**Study participant selection.** ILAS = I-Lan Longitudinal Aging Study; MMSE = Mini-Mental State Examination.

Among 1281 ILAS participants with qualifying brain MRI images, 85 were excluded due to brain abnormalities (infarct, haemorrhage, tumour), head motion or artefacts, dementia, or missing data; the analytic sample comprised 1196 subjects ≥ 50-year-olds who had no dementia or evident physical disabilities (62 ± 9 years old, 47.6% men). Quality screening disqualified a further 190 candidates from subsequent tractography analyses (1196 for GMV analyses and 1006 for tractography analyses; Figure 1).

Table 1 shows the comparisons of demographics, clinical characteristics and MRI results between 1006 non-PCDS participants (84.1%) and 190 subjects with PCDS (15.9%). There were 155 (13.0%) subjects who had mobility components of physical frailty but without cognitive impairment. The PCDS group was significantly older and more prevalent female sex and diabetes mellitus (Table 1).

**Table 1:** Demographics and clinical characteristics.

	**All (n = 1196)**	**Non-PCDS (n = 1006)**	**PCDS (n = 190)**	***P* value**
Age, years	62 ± 9	62 ± 9	65 ± 9	<0.001
Male sex, %	47.6	49.0	40.0	0.026
Hypertension, %	38.3	37.2	44.2	0.073
Diabetes, %	14.9	13.3	23.2	0.001
Hyperlipidemia, %	6.9	6.5	8.9	0.212
Smoking, %	16.9	16.5	18.9	0.286
BMI, kg/m^2^	24.6 ± 3.3	24.6 ± 3.3	24.6 ± 3.5	0.972
Education, years	6.9 ± 5.1	7.2 ± 5.0	5.2 ± 4.9	<0.001
Centre for Epidemiologic Studies Depression Scale	2.2 ± 4.2	2.0 ± 3.7	3.3 ± 6.2	0.004
Mini-Mental State Examination, point	26.5 ± 3.3	26.8 ± 3.0	24.7 ± 4.2	<0.001
Impaired cognitive domains, %				
10-minute CVVLT	10.4	8.7	18.9	<0.001
Boston naming test	10.1	7.7	23.2	<0.001
Verbal fluency test	8.3	5.9	21.1	<0.001
Taylor complex figure test	6.4	4.4	17.4	<0.001
Backward digit test	21.1	15.5	50.5	<0.001
Clock drawing test	7.9	6.3	16.8	<0.001
Total intracranial volume, cm^3^	1448.5 ± 127.9	1452.8 ± 125.6	1450.8 ± 138.5	0.808^a^
Gray-matter volume, cm^3^	596.9 ± 54.4	597.9 ± 52.8	591.5±59.4	0.001^b^
White-matter volume, cm^3^	465.3 ± 56.1	466.0 ± 54.5	461.2 ± 62.1	0.015^b^

^a^Analysis of covariance adjusted for age and sex.

^b^Analysis of covariance adjusted for age, sex and total intracranial volume.

PCDS = physio-cognitive decline syndrome; BMI = body mass index; CVVLT = Chinese version Verbal Learning Test.

Table 2 demonstrates the comparisons between non-PCDS subjects and PCDS subjects in groups < 65 and ≥ 65 years old respectively. Individuals with PCDS in both age groups were significantly older, less educated and had lower mini-nutrition assessment scores. Compared with non-PCDS subjects, participants with PCDS had significantly lower scores and higher frequencies of impairment in each cognitive domain in both middle-aged (< 65 years old) and elderly (≥ 65 years old) groups. Subjects with PCDS had higher Center for Epidemiologic Studies Depression Scale scores than non-PCDS subjects, however, the difference was only statistically-significantly in ≥ 65-year-olds. The PCDS group also had a higher prevalence of cardiovascular risk factors, particularly diabetes in the middle-aged and coronary artery disease in the elderly.

**Table 2:** Demographics and clinical characteristics of middle-aged and old subjects.

	**Middle-aged (< 65 years old)**			**Old-aged (≥ 65 years old)**		
	**Non-PCDS**	**PCDS**	***P* value**	**Non-PCDS**	**PCDS**	***P* value**
N (%)	671 (84.9)	119 (15.1)		335 (82.5)	71 (17.5)	
Age, years	56 ± 4	58 ± 4	<0.001	72 ± 5	75 ± 6	<0.001
Male sex, %	44.4	41.2	0.548	58.2	38.0	0.002
Hypertension, %	30.1	36.1	0.198	51.3	57.7	0.361
Diabetes, %	11.8	21.0	0.012	16.4	26.8	0.061
Hyperlipidemia, %	6.1	10.9	0.073	7.2	5.6	0.800
Smoking, %	15.4	21.8	0.121	18.8	14.1	0.321
BMI, kg/m^2^	24.7 ± 3.3	24.8 ± 3.8	0.661	24.4 ± 3.1	24.1 ± 2.9	0.549
Education, years	8.9 ± 4.3	7.3 ± 4.7	<0.001	3.9 ± 4.7	1.8 ± 2.9	<0.001
Center for Epidemiologic Studies Depression Scale	1.8 ± 3.8	2.9 ± 5.8	0.064	2.2 ± 3.6	4.1 ± 6.7	0.025
Mini-Mental State Examination, point	27.9 ± 2.0	26.2 ± 3.4	<0.001	24.7 ± 3.4	22.1 ± 4.2	<0.001
Impaired cognitive domains, %						
10-minute CVVLT	8.5	18.5	0.002	9.3	19.7	0.020
Boston naming test	6.6	25.2	<0.001	9.9	19.7	0.024
Verbal fluency test	6.1	25.2	<0.001	5.4	14.1	0.017
Taylor complex figure test	4.2	16.0	<0.001	4.8	19.7	<0.001
Backward digit test	9.8	37.0	<0.001	26.9	73.2	<0.001
Clock drawing test	5.8	17.6	<0.001	7.2	15.5	0.034
Total intracranial volume, cm^3^	1459.8 ± 126.3	1457.4 ± 140.9	0.819	1439.1 ± 124.2	1441.1 ± 133.6	0.883^a^
Gray-matter volume, cm^3^	612.7 ± 49.8	607.9 ± 52.3	0.042	568.9 ± 47.9	558.7 ± 51.4	0.003^b^
White-matter volume, cm^3^	478.1 ± 52.4	474.3 ± 59.8	0.106	442.5 ± 51.7	434.3 ± 53.0	0.018^b^

^a^Analysis of covariance adjusted for age and sex.

^b^Analysis of covariance adjusted for age, sex and total intracranial volume.

PCDS = physio-cognitive decline syndrome; CVVLT = Chinese version Verbal Learning Test.

Brain volumetry analyses also showed differences between PCDS and non-PCDS groups. Although total intracranial volumes (TIV) were comparable, subjects with PCDS in both aged groups had significantly lower GMV and higher cerebrospinal fluid volume. In the elderly group, PCDS subjects also had significantly lower white matter volume than non-PCDS subjects.

### Neuroanatomic correlates of PCDS

**Figure 2:**
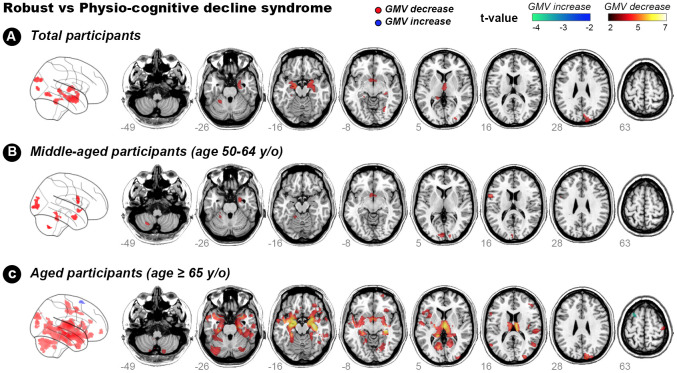
**Hot colour map of gray-matter volume-diminished regions in subjects with physio-cognitive decline syndrome.** GMV = gray-matter volume; L = left; R = right; PCDS = physio-cognitive decline syndrome.

Voxel-based whole-brain analysis showed that participants with PCDS had lower GMV than non-PCDS ones in right and left amygdala, right and left thalamus, right hippocampus, right temporal occipital fusiform cortex, right occipital pole, and left cerebellum VI and V (Figure 2A and Table 3, family-wise error cluster corrected P-value < 0.05). The regions with GMV decrement in PCDS subjects were similar between the middle-aged and older adults; whereas larger clusters with more extensive GMV-decrement regions were observed in ≥ 65-year-olds with PCDS than in non-PCDS contemporaries (Figure 2B, 2C and Table 4, family-wise cluster corrected P-value < 0.05).

**Table 3:** Reduced gray-matter volume in physio-cognitive decline syndrome subjects.

**Cluster size**	**Anatomical region**		**MNI-space coordinates**			**Maximum intracluster t value**
	**Side**	**Structure**	**X**	**Y**	**Z**	
1883	Right	Amygdala	21.0	0	-17.0	4.18
Right	Hippocampus	27.0	-12.0	-19.5	4.10
818	Left	Amygdala	-16.5	-10.5	-13.5	4.85
535	Right	Thalamus	1.5	-7.5	9.0	3.85
472	Right	Occipital pole	14.0	-93.5	30.0	4.00
379	Left	Cerebellum VI	-24.0	-47.3	-25.5	3.58
Left	Cerebellum V	-25.5	-39.0	-19.5	3.33
241	Left	Thalamus	-22.5	-33.0	-1.5	3.77
182	Right	Temporal occipital fusiform cortex	31.5	-58.5	-9.8	3.88

Analyses adjusted for age, sex, Centre for Epidemiologic Studies Depression Scale and cardiovascular risk factors (hypertension, diabetes, hypderlipidemia, smoking and obesity).

MNI = Montreal Neurological Institute.

**Table 4:** Reduced gray-matter volume in physio-cognitive decline syndrome subjects of two aged groups.

**Cluster size**	**Anatomical region**		**MNI-space coordinates**			**Maximum intracluster t value**
	**Side**	**Structure**	**X**	**Y**	**Z**	
**Reduced GMV in PCDS < 65 years old**						
598	Left	Occipital pole	-13.5	-100.5	0	3.99
405	Left	Precentral gyrus	-58.5	7.5	15.0	4.32
298	Right	Amygdala	25.5	0	-27.0	3.69
255	Left	Cerebellum V	-18.0	-45.0	-16.5	3.40
138	Left	Cerebellum VIIIa	-24.8	-62.3	-47.3	4.38
**Reduced GMV in PCDS ≥ 65 years old**						
30986	Right	Hippocampus	32.3	-15.8	-10.5	6.97
	Right	Thalamus	1.5	-12.0	-10.5	6.51
Left	Amygdala	-27.0	-12.0	-15.0	6.19
2580	Right	Cerebellum Crus I	30.8	-66.0	-33.0	4.29
2013	Left	Cerebellum Crus I	-31.5	-69.0	-31.5	4.37
1274	Right	Occipital pole	13.5	-91.5	30.0	4.68
869		Cerebellum vermis VIIIa	4.5	-67.5	-39.0	3.99
598	Right		58.0	-25.5	16.0	4.14
584	Right	Postcentral gyrus	45.0	-19.5	63.0	3.90
562	Right	Lateral occipital cortex	52.5	-63.0	13.5	4.32
545	Right	Cingulate gyrus	7.5	1.5	40.5	4.23

Analyses adjusted for age, sex, Centre for Epidemiologic Studies Depression Scale and cardiovascular risk factors (hypertension, diabetes, hypderlipidemia, smoking and obesity).

MNI = Montreal Neurological Institute. GMV = gray-matter volume.

### Connections between cerebellum and hippocampus/amygdala

**Figure 3:**
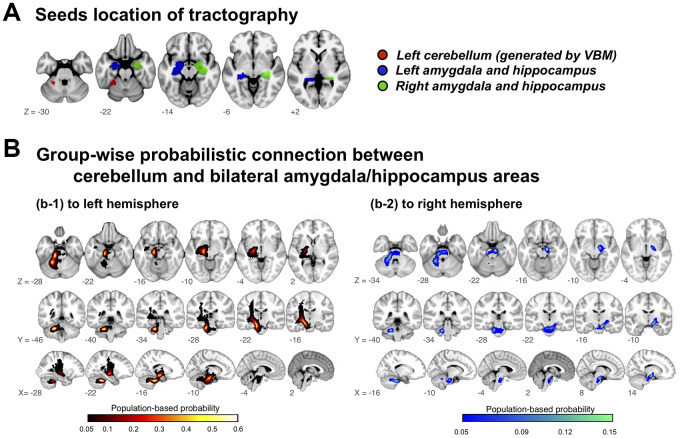
**Group-wise probabilistic connections between cerebellar and bilateral amygdala/hippocampus areas.** VBM = voxel-based morphometry.

GMV in left cerebellum was decreased in subjects with PCDS of both age groups. This is in consistent with our previous study which showed an association between reduced cerebellum GMV and physical frailty [17]. The present study also identified decreased hippocampus/amygdala GMV in subjects with PCDS, which has long been recognized as an early marker of cognitive impairment in the elderly [23]. Due to a close relationship between physical and cognitive declines in the elderly, we postulated a neurocircuit connecting left cerebellum and hippocampus/amygdala which might be involved in the pathophysiology of PCDS (Figure 3A). We used diffusion-weighted tractography aiming to establish a population-based probability map of our hypothetical neuroanatomical connections.

Among 1006 included participants, delineated tracts successfully connected cerebellar regions of interest to the left and right hippocampus/amygdala area in 72.5%, but to the right amygdala/hippocampus area in only 20.8%; the tract probability map (likelihood of participant’s cerebellar-hippocampus/amygdala connection passing through the voxel) described a highly coherent connection path across participants, with maximal probability of 60.8%/72.5% on the left side and 15.0%/20.8%) on the right (Figure 3B).

### Cerebellum-hippocampus/amygdala tract abnormalities in PCDS

Tract-specific analyses showed that within bilateral cerebellum to amygdala/hippocampus connections, PCDS subjects had higher values than non-PCDS ones in three different diffusivity indices (mean, radial and axial) (Table 5, P-value < 0.05). However, differences between non-PCDS subjects and those with PCDS in tract-specific analyses for altered fractional anisotropy were not statistically significant.

**Table 5:** Tract-wise diffusivity differences between non-physio-cognitive decline syndrome subjects and those with physio-cognitive decline syndrome.

	**Total**	**Non-PCDS**	**PCDS**	***p* value**
N (%)	1006	845 (84.0%)	161 (16.0%)	
Left connection				
Fractional anisotropy	0.44 ± 0.02	0.44 ± 0.02	0.43 ± 0.02	0.713
Mean diffusivity (10^−3^ mm^2^ s^−1^)	0.89 ± 0.05	0.89 ± 0.05	0.91 ± 0.06	0.008
Radial diffusivity (10^−3^ mm^2^ s^−1^)	0.69 ± 0.05	0.69 ± 0.05	0.70 ± 0.06	0.014
Axial diffusivity (10^−3^ mm^2^ s^−1^)	1.30 ± 0.05	1.30 ± 0.05	1.32 ± 0.06	0.009
Right connection				
Fractional anisotropy	0.44 ± 0.02	0.44 ± 0.02	0.44 ± 0.03	0.310
Mean diffusivity (10^−3^ mm^2^ s^−1^)	0.87 ± 0.07	0.87 ± 0.07	0.89 ± 0.09	0.004
Radial diffusivity (10^−3^ mm^2^ s^−1^)	0.66 ± 0.07	0.66 ± 0.07	0.68 ± 0.09	0.006
Axial diffusivity (10^−3^ mm^2^ s^−1^)	1.29 ± 0.07	1.30 ± 0.07	1.32 ± 0.09	0.003

Analyses adjusted for age, sex, Centre for Epidemiologic Studies Depression Scale and cardiovascular risk factors (hypertension, diabetes, hypderlipidemia, smoking and obesity).

PCDS = physio-cognitive decline syndrome.

### Correlations between GMV in PCDS-associated brain regions and each cognitive/physical domain

**Figure 4:**
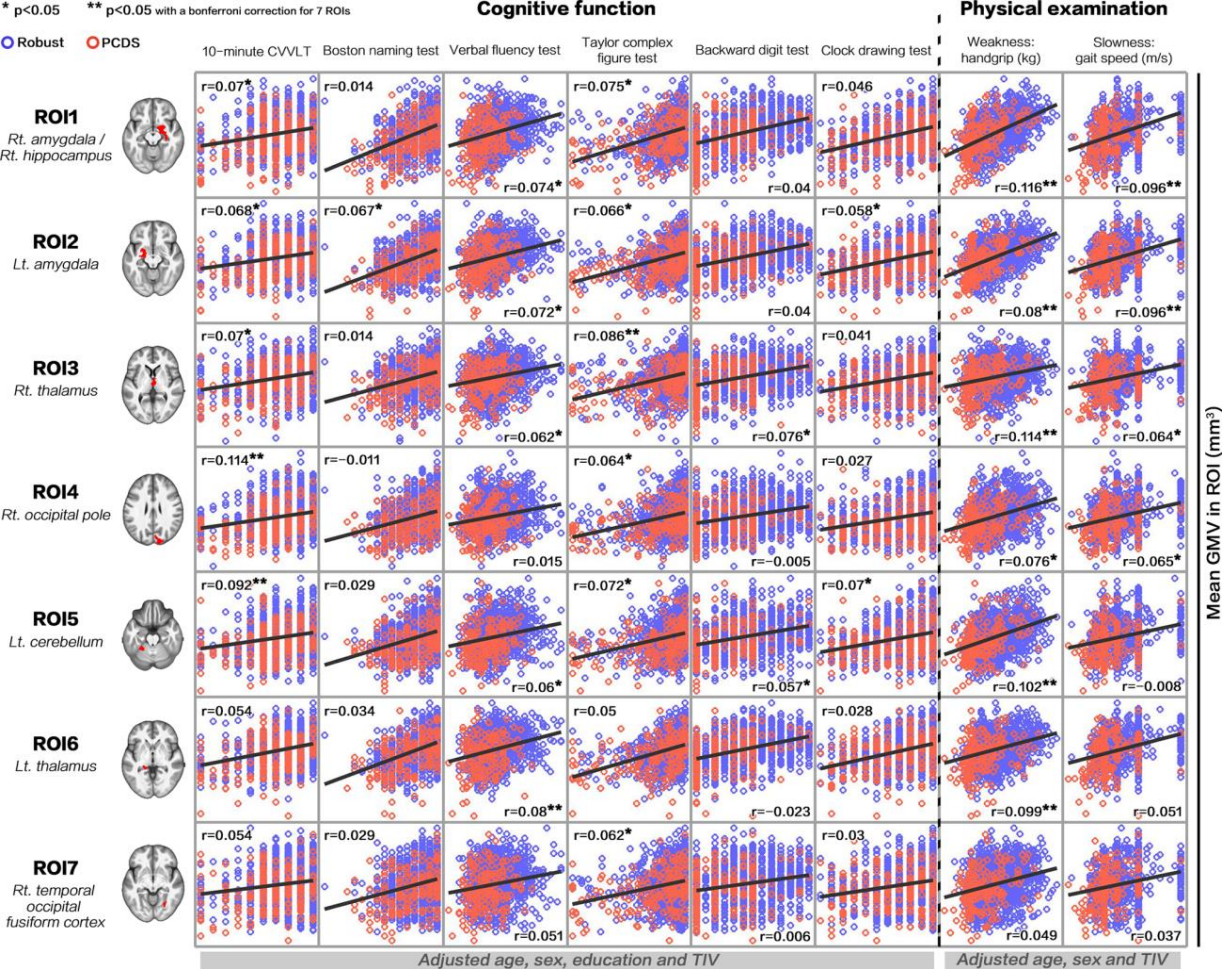
**Correlations between gray-matter volume in physio-cognitive decline syndrome-associated brain regions and each cognitive/physical domain.** PCDS = physio-cognitive decline syndrome; ROI = region of interest; Rt. = right; Lt. = left; CVVLT = Chinese version Verbal Learning Test.

The results showed positive correlations between GMV in PCDS-associated brain regions and each cognitive (age, sex, education, TIV-adjusted) and physical (age, sex, TIV-adjusted) performances respectively (Figure 4). Statistically significances after Bonferroni correction for 7 regions of interests (ROIs) were shown in right amygdala/hippocampus (versus handgrip strength and gait speed), left amygdala (versus handgrip strength and gait speed), right thalamus (versus visuospatial function and handgrip strength), right occipital pole (versus verbal memory), left cerebellum (versus verbal memory and handgrip strength) and left thalamus (versus verbal fluency and handgrip strength).

## DISCUSSION

The present study discovered neuroanatomic deficits and the postulated neurocircuit associated with PCDS. PCDS was significantly associated with (1) reduced GMV in amygdala, thalamus, hippocampus, temporo-occipital cortex, and cerebellum VI and V regions and (2) disrupted hippocampus-amygdala-cerebellum connections. These findings may provide clues to the early neuroanatomic pathognomonic of age-related concomitant physical and cognitive decline. This neuroanatomic process was noted as early as middle-age; subjects with PCDS aged 50–64 years already had GMV decrement in the cerebellum, occipital cortex and amygdala. Notably, PCDS appeared to originate from the left side; clusters of GMV decrement in middle-aged subjects expanded and extended into the right side until ≥ 65 years old. The finding with reduced cerebellar GMV in PCDS was consistent with our previous study, which showed an association between reduced cerebellum GMV and physical frailty, particularly its motor-related components [17]. Hippocampal atrophy is a hallmark of Alzheimer’s disease and manifests early in prodromal MCI [23]. However, as cerebellar atrophy is not typical of early-stage Alzheimer’s disease, our results suggest that Alzheimer’s pathology may not be fully accountable to the cognitive impairment in PCDS.

Clinical and animal studies have identified cerebellar functions beyond motor control [24, 25]. Functional neuroimaging has linked the cerebellum to cognitive domains including spatial processing, working memory, and executive functions [24, 26]. We also showed that, in addition to handgrip strength (weakness), multiple cognitive domains including verbal memory, verbal fluency, visuospatial function and executive functions were positively associated with left cerebellar GMV (Figure 4). We thus postulate that in addition to physical frailty [17], cognitive impairment in early aging might also be ascribed to cerebellar deficits [27], which would make the cerebellum the principal area responsible for maintaining both physical and cognitive functions in aging. However, further functional neuroimaging studies are needed to confirm this hypothesis.

In addition to mapping the neuroanatomic regions involved in PCDS, we also delineated a connecting neurocircuit. Using diffusion-weighted tractography, we have delineated tracts connecting the left cerebellum to bilateral hippocampus/amygdala, with a left-sided preponderance. Abnormal diffusion-tensor imaging indices within this tract suggest that this neurocircuit is involved in PCDS. Tracking cerebellar networks is challenging due to their tortuous pathways inside and outside the cerebellum. Nevertheless, a recent diffusion-tensor imaging study successfully reconstructed tracts from the cerebellum to the cerebral cortex of each lobe [28]. Like our results showing a predominant ipsilateral and left side cerebellum-hippocampus/amygdala connection, they also revealed that temporo-ponto-cerebellar tract, unlike fronto-ponto-cerebellum tract, has larger volume on the left side and with unilateral connection (uncross to contralateral side) [28]. Connections between the cerebellum and hippocampus/amygdala were first detected by animal neurology studies [29]. Later clinical research focused on neuropsychiatric disorders, such as emotional dysregulation and depression, and described this connection as part of the cortico**-**limbic-cerebellar circuit [30, 31]. We are the first to provide evidence that this connection is involved in early concomitant age-related physical and cognitive decline. Notably, rather than cerebellar vermis, which has a regulatory role in the limbic system [32], the junction between the left anterior and posterior lobes was associated with PCDS. We postulate that PCDS might emanate from the left cerebellum and its connection to the hippocampus/amygdala, and that this neurocircuit is a potential target for prevention and treatment trials.

In contrast with the clinical associations of MCRS, studies about the neuroimaging characteristics of MCRS are scarce. One France study (GAIT cohort study) with 171 community-dwelling elderly (16.4% of them were MCRS) showed a significantly reduced total, prefrontal and motor cortex GMV in subjects with MCRS [33]. Another study jointed the same cohort with the other two cohorts in the US (total population = 267, 14.2% MCRS) identified a significant GMV covariance pattern that was associated with MCR; this GMV covariance network was primarily composed of supplementary motor, insular and prefrontal cortex regions [34]. Similar to our results from PCDS, cerebellar and parahippocampal regions have also been found involving in this MCR-related network [34]. The motor and control aspects of gait, e.g. initiation/maintenance and planning/modulation, both originate in the frontal cortex [35–37]. This pathway would need the integration from the cerebellum before conveying information to the spinal cord and also the feedback information via the cerebellum [35–37]. In the present study, the associated GMV reduction in PCDS, however, spared the frontal cortex. The frontal lobe itself is vulnerable to aging process [38]. Several brain structural imaging studies have confirmed age-related, progressive total and regional brain atrophy since middle age [38]. Among all brain regions, frontal lobe showed the greatest decline with age and its atrophy accelerated at a more advanced age [38]. Neuroimaging studies in MCRS (mean age: 70 and 75 years old) included older subjects than the present study (mean age: 60 years old) [33, 34]. The present results in population with a relatively younger age might represent an earlier stage of age-related physio-cognitive decline. Therefore, in addition to the diverse definitions of cognitive frailty which might reflect different underlying pathogenesis, the discrepancy may be due to different disease stages between MCRS and PCDS. Future neuroimaging studies with a larger study population involving different races and longitudinal follow-up warrant investigations.

The strengths of our study include its large sample size and age range, from middle-aged to elderly. In addition to age and sex, our analyses were also adjusted for cardiovascular risk factors which were more prevalent in our PCDS cohort and are known determinants of GMV diminution. However, our results should be interpreted in light of some limitations. First, ILAS participants live in rural communities; due of their background and environment, they were relatively physically fit and less well educated. The generalizability of our results to populations with different demographics deserves further study. In addition, PCDS subjects in the present study had prevalent vascular risk factors which might also contribute to the neuroanatomic and neurocircuit findings. However, GM atrophy in hypertension and diabetes are located in frontal or/and temporal lobes [39–41] instead of posterior brain regions including cerebellum which has been shown atrophy in subjects with PCDS. Most importantly, we have adjusted these vascular risk factors during analyses to mitigate potential confounder effects. Secondly, the current approach (PCDS versus non-PCDS) could not reveal how each of the components of PCDS (physical frailty, cognitive impairment or certain cognitive domains) contributed to the neuroanatomic findings. A consequential study will be conducted to address this concern and the results would further provide mechanistic insights into the relationship between abnormal neuroanatomic/neurocircuit findings and PCDS. Lastly, the cross-sectional design of this study precluded establishing causality; a future longitudinal study could not only evaluate the causal role of the neuroanatomic features and neurocircuit in PCDS but also explore the temporal neuroanatomic changes as PCDS progresses.

In conclusion, brain imaging studies of this large community-based cohort revealed characteristic neuroanatomic and neurocircuit features associated with PCDS, a phenotype of early physical and cognitive impairment in the elderly. The left cerebellum and its connections to the hippocampus/amygdala might be involved in the early neural pathogenesis and mediate physical and cognitive decline in aging.

## MATERIALS AND METHODS

### Study population

Participants were selected from the ILAS cohort, which was established to study the interrelationships between aging, frailty, and cognition, and comprises 1,839 community-dwelling adults aged ≥ 50 years. ILAS excluded people who: were unable to communicate and complete an interview, or unable to complete assessments due to poor functional status; had life expectancy < 6 months due to major illnesses; had any contraindication for MRI, such as metal implants; and were institutionalized. This imaging study further excluded ILAS subjects with major organic brain disorders such as a stroke or tumor. All participants gave signed informed consent. The Institutional Review Board of National Yang Ming University, Taipei, Taiwan, approved the study.

### Demographics, physical examinations, and laboratory measurements

Participants completed a questionnaire to elicit their demographic information, cigarette smoking habits, and medical history. Functional assessments included the Functional Autonomy Measurement System (physical function), the Center for Epidemiologic Studies Depression Scale (mood status), and the Mini-Nutrition Assessment (nutritional status). Additional physical and laboratory measurements are available in Supplementary Material.

### Cognitive function assessment and definition of dementia

All participants underwent a face-to-face neuropsychological examination administered by trained interviewers. Global cognitive performance was firstly assessed using the Mini-Mental State Examination (MMSE). Since MMSE performance largely depends on educational status, a population-based study in Taiwan determined the MMSE cut-offs according to different education years for correlation with dementia, diagnosed according to DSM-III-R criteria [42, 43]. Consequently, epidemiological studies of the Taiwan population, including ILAS have defined dementia as an MMSE score < 24 in well-educated subjects (education years ≥ 6) or < 14 in less-educated subjects (education years < 6) [43]. ILAS also used this definition of dementia, and this study excluded subjects who met these criteria.

Participants also had comprehensive neuropsychological assessments across multiple cognitive domains. Cognitive impairment in each domain was defined as a score in each test below 1.5 standard deviations of age- and education-matched norms for the same population. Detailed method of neuropsychological assessment is available in Supplementary Material.

### Definition of PCDS

Mobility component of physical disability, i.e. weakness and slowness, was used to define age-related declines in physical function [44]; either grip strength (weakness component) or walking speed (slowness component) below cut-offs proposed by Asian Working Group for Sarcopenia was considered declines in physical function [45]. The cutoff value for handgrip strength were <26 kg for men and <18 kg for women, for gait speed was <0.8 m/s. PCDS was defined as declines in physical function concurrent with cognitive impairment in any domain, but without diagnosed dementia or physical disability.

### Neuroimaging studies

All MRI data were acquired using a single 3T imaging system with a 12-channel head coil (Siemens Magnetom Tim Trio, Erlangen, Germany). All images were acquired along the anterior/posterior commissure line without in-plane interpolation and inter-slice gap; head movement was minimized using foam pads. An experienced neuroradiologist inspected all anatomical scans before further analysis to exclude any with motion artefacts or gross brain abnormalities, including trauma, tumours, and haemorrhagic or infarct lesions.

Anatomical MRI scans to estimate tissue volume, identify white matter lesions and for tractography reconstruction, entailed: (1) a sagittal three-dimensional T1-weighted magnetization-prepared rapid-acquisition gradient echo sequence (3D-T1w-MPRAGE: repetition time/echo time/ inversion time = 3500/3.5/1100 ms; flip angle = 7°; number of excitations = 1; field of view = 256 × 256 mm^2^; matrix size = 256 × 256; 192 slices, with voxel size = 1.0 × 1.0 × 1.0 mm^3^); (2) an axial two-dimensional T2-weighted fluid-attenuated inversion-recovery multi-shot turbo-spin echo sequence (2D-T2w-FLAIR BLADE: repetition time/echo time/inversion time = 9000/143/2500 ms; flip angle = 130°; number of excitations = 1; field of view = 220 × 220 mm^2^; matrix size = 320 x 320, echo train length = 35; 63 slices, with voxel size = 0.69 × 0.69 × 2.0 mm^3^); and (3) an axial single-shot spin-echo echo-planar imaging diffusion-weighted sequence (repetition time/echo time = 11000/104 ms; number of excitations = 3; field of view = 256 × 256 mm^2^; matrix size = 128 × 128; 70 slices, with voxel size = 2.0 x 2.0 x 2.0 mm^3^; b value = 1000 s/mm^2^; 30 non-collinear gradient directions and three non-diffusion-weighted T2 images). The origin of each scan was re-oriented, using an automated centre-of-mass approach, to further minimize variability in scanning position and errors in subsequent image registration and segmentation.

### Voxel-wise gray-matter volume (GMV) estimation

We used voxel-based morphometry (VBM) to investigate GMV changes between study groups [46]. All native-space re-oriented T1w and T2w-FLAIR scans were pre-processed using the Computational Anatomy Toolbox (CAT12, version 1266 http://www.neuro.uni-jena.de/cat/), the Lesion Segmentation Toolbox (LST, version 2.0.15, http://www.applied-statistics.de/lst.html) [47] and Statistical Parametric Mapping software (SPM12, version 6909, Wellcome Institute of Neurology, University College London, UK, http://www.fil.ion.ucl.ac.uk/spm/) in Matlab R2016a (The Mathworks, Inc., Natick, MA, USA) with default settings. Our VBM pre-processing procedure was the same as previously described [17]: (1) individual T2w-FLAIRs were affine registered to corresponding T1w anatomical scans using the “Coregister” function of SPM12; (2) using LST, co-registered T2w-FLAIR T1w scans served as inputs for localizing and refilling white matter lesions, with intensities similar to the normal white matter appearance for each individual; (3) lesion-filled T1w scans were corrected for non-uniform intensities, and segmented into gray matter, white matter and cerebrospinal fluid tissue maps using the CAT12 toolbox; (4) using the high-dimensional Diffeomorphic Anatomical Registration Exponentiated Lie Algebra (DARTEL) warping algorithm [48], gray and white matter tissue maps were iteratively registered to create study-specific Montreal Neurological Institute (MNI)-space tissue templates based on the entire study cohort; (5) the deformation field from DARTEL normalization was applied to warp corresponding gray matter segments into the MNI space; (6) voxel-wise estimates of GMV from these MNI-space gray matter tissue maps were obtained by modulating the linear and non-linear components of the Jacobian determinant of estimated deformation fields; (7) finally, MNI-space modulated gray matter segments were smoothed by convolution with an 8 mm full-width at half-maximum, isotropic Gaussian kernel. The final spatial resolution of MNI-space modulated gray matter tissue segments was 1.5 mm cubic/voxel.

The normalized, modulated and smoothed gray matter tissue segments were used for subsequent statistical analyses. After completing VBM pre-processing, the CAT12 toolbox “Check Data Quality” module was used to check the data homogeneity of pre-processed gray matter segments; no subjects were considered outliers and excluded. To adjust for global variation in brain size, each participant’s gray matter, white matter, and cerebrospinal fluid volumes in native space were added to give the total intracranial volume, which served as a confounding factor in statistical analyses. To minimize the potential partial volume effect, individual MNI-space unmodulated gray matter tissue maps were averaged at an intensity threshold 0.2, to create an explicit binary mask for subsequent voxel-wise analyses.

### Post-hoc reconstruction of hypothetical tractography in the cerebellum and bilateral amygdala-hippocampal regions

Due to a close relationship between physical and cognitive declines in older adults [11, 49], we hypothesised that a neurocircuit may underlie PCDS and used diffusion-weighted tractography to map putative connections between the left cerebellum and hippocampus/amygdala. Prior to tractography analysis, an experienced neuroradiologist inspected all diffusion-weighted images to further exclude those with unacceptable motion or other artefacts. Probabilistic tractography of pre-defined regions of interest was then used to investigate possible neuroanatomic connections between the cerebellum and bilateral amygdala-hippocampal regions using MRtrix3 (http://www.mrtrix.org/) and FMRIB Software Library (FSL, version 5.0.11, http://fsl.fmrib.ox.ac.uk/fsl/fslwiki/). Cerebellar regions of interest were identified based on VBM results, while those in the amygdala and hippocampus were identified by the automated anatomical labelling atlas [50], and merged into a single region of interest for each hemisphere. Initial tractography was first performed in the native diffusion space for each individual and further warped into the standard MNI space, to produce a population-based probability map of our postulated neuroanatomical connections.

Tractography entailed four steps: (1) individual diffusion-weighted scans were affine-aligned to corresponding non-diffusion-weighted scans, to correct for head movement and eddy current distortion, then skull-stripped using the Brain Extraction Tool [51]; (2) to constrain subsequent analyses within white matter areas, 5ttgen (a MRTrix command-line tool that integrates the Brain Extraction Tool, and FSL's Automated Segmentation Tool [52] and Integrated Registration and Segmentation Tool [53]) was used to segment individual T1w anatomical scans into white matter, grey matter, cerebrospinal fluid, subcortical regions, and diseased tissue compartments; (3) using the MRtrix3 command-line tools dwi2response and dwi2fod [54, 55], the voxel-wise fibre orientation density function of each individual was computed in native diffusion space; (4) a two-stage registration approach [56] was used to transform the pre-identified MNI-space regions of interest into native diffusion space. The boundary-based registration capability of FSL’s Linear Image Registration Tool [57] was first used to calculate the transformation matrix between non-diffusion-weighted images and corresponding T1w anatomical scans. The second stage transformation matrix was determined by registering each T1w scan to the standard MNI T1 template with a nonlinear registration approach using FSL’s Non-Linear Image Registration Tool [58]. To minimize interpolation artefacts, we further merged these two transformation matrices into a single matrix for each individual for spatial transformation of regions of interest; (5) finally, anatomically-constrained tractography [59] using the iFOD2 [32] algorithm was applied to reconstruct hypothetical tracts connecting cerebellar regions of interest to bilateral amygdala-hippocampal regions; the seed and target maps were exchangeable between cerebellum and amygdala/hippocampus, where 20 seeds/voxel were randomly planted in native diffusion space. Tracts were depicted along the fibre orientation density function with a 0.5 mm step-size, maximum 60° turning angle, and minimum 0.1 amplitude. Having established putative tracts, we first merged bi-directional tracts between cerebellum and amygdala/hippocampus, then converted them into binary map, where 1 represents the tract intersecting the voxel. Finally, individual binary tract maps were warped into the standard MNI space. By summing the information from each participant, normalized by the total participant number, the final tractography image represents the proportion of subjects in whom each pathway was present.

### Extracting multiple quantitative diffusivity indices of hypothetical anatomical connections

To investigate potential microstructural differences between study groups in the hypothetical cerebellum to amygdala/hippocampus connections, MNI-space population-based probability maps were used as templates for extracting multiple diffusivity indices in native space. First, we used the command-line tool DTIFIT (available in FSL) to estimate the voxel-wise fractional anisotropy, mean diffusivity, radial diffusivity, and axial diffusivity for each participant. Then, the MNI-space population-based probability map was spatial transformed into individual native space to extract probability-weighted averages for the multiple diffusivity indices of each hypothetical anatomical connection [60].

### Statistical analyses

All statistical analyses used SPSS Statistics for Windows version 19.0 (IBM Corp. Released 2010. IBM SPSS Statistics for Windows, Version 19.0. Armonk, NY: IBM Corp.). Continuous variables were expressed as mean plus/minus standard deviation, categorical variables as proportions. Continuous data from PCDS versus non-PCDS groups were compared by Student’s t test; categorical data were compared by Chi-square analysis, as appropriate. P < 0.05 was considered statistically significant.

GMV and diffusivity differences between non-PCDS and PCDS groups were determined using a voxel-wise single-factor two-level ANCOVA procedure with confounding factors of age, sex, total intracranial volume, Center for Epidemiologic Studies Depression Scale score, and cardiovascular risks including history of hypertension, diabetes, dyslipidaemia, smoking, and obesity (BMI > 30 kg/m^2^). The same statistical design was also applied to the multiple quantitative diffusivity indices of the hypothetical anatomical connections to identify the between group difference (PCDS versus non-PCDS groups) in white matter microstructural properties.

We further stratified the analytic cohort into 50–64-years-olds (middle-aged) and ≥65-year-olds (elderly) to investigate potential effects of age on analytic GMV results. The GLM_Flex toolbox (http://mrtools.mgh.harvard.edu/index.php?title=GLM_Flex) was used to identify the anatomical correlates (GMV) of PCDS, voxel-wise, in different age-groups.

The significance level for all voxel-wise analyses was set at cluster-level family-wise error corrected P-value < 0.05 with a cluster-forming threshold of voxel-level P-value < 0.001 and 113 voxel extents, determined from results of Monte Carlo simulation with the command-line tool 3dClustSim (10000 permutations with explicit GM mask, version AFNI_18.1.18). Statistical results for clusters with significant between-group main effects, including cluster sizes, maximum Z-values, anatomical locations and corresponding MNI coordinates were reported using the Peak_nii toolbox (https://www.nitrc.org/projects/peak_nii).

To investigate the potential associations between observed neuroanatomical alterations and each cognitive/physical task in our population, PCDS-associated anatomical regions identified by the above voxel-wise analysis were extracted and averaged for each individual. Partial Pearson’s correlation analyses which adjust corresponded confounding factors (for domains of cognitive functions: age, sex, education and TIV adjusted; for physical examinations: age, sex and TI adjusted) were then used to identify the potential associations between regional GMV measures and multiple domains of cognitive functions (including scores of 10-minute CVVLT, Boston naming test, verbal fluency test, Taylor complex figure test, backward digit test, and clock drawing test) and physical examinations (including handgrip and gait speed) respectively. The statistical significances were reported both with the threshold at an uncorrected P-value<0.05 and a Bonferroni corrected P-value<0.05 (for seven regions).

## Supplementary Material

Supplementary Materials
