# Supplementary material for: Cerebellar-limbic neurocircuit is the novel biosignature of physio-cognitive decline syndrome
Source: Aging (Albany NY). 2020 Nov 25;12(24):25319–36. doi: 10.18632/aging.104135 (PMC7803525; doi:10.18632/aging.104135)
Supplement: Supplementary Materials [file aging-12-104135-s001.pdf]

SUPPLEMENTARY MATERIALS

Supplementary Methods

Demographics, physical examinations, and laboratory measurements

Participants completed a questionnaire to elicit their demographic information, cigarette smoking habits, and medical history. Functional assessments included the Functional Autonomy Measurement System (physical function), the Center for Epidemiologic Studies Depression Scale (mood status), and the Mini-Nutrition Assessment (nutritional status).

Research nurses took anthropometric measurements including waist circumference, and height and body weight to derive the body mass index (BMI). Grip strength of the dominant hand was measured using digital dynamometry (Smedlay's Dynamo Meter; TTM, Tokyo, Japan); participants stood with their arms by their sides and the best of three readings was used for analysis. The time each subject took to walk 6 metres was measured to assess physical performance. Body composition was determined by whole body dual-energy X-ray absorptiometry using a Lunar Prodigy scanner (GE Healthcare, Madison, WI, USA). Appendicular skeletal muscle mass was calculated as the total lean mass of four limbs, then divided by height (in metres) squared to derive the relative appendicular muscle mass (kg/m^2).

Cognitive function assessment and definition of dementia

All participants underwent a face-to-face neuropsychological examination administered by trained interviewers. Global cognitive performance was firstly assessed using the Mini-Mental State Examination (MMSE). Since MMSE performance largely depends on educational status, a population-based study in Taiwan determined the MMSE cut-offs according to different education years for correlation with dementia, diagnosed according to DSM-III-R criteria [1, 2]. Consequently, epidemiological studies of the Taiwan population, including ILAS have defined dementia as an MMSE score < 24 in well-educated subjects (education years ≥ 6) or < 14 in less-educated subjects (education years < 6) [2]. ILAS also used this definition of dementia, and this study excluded subjects who met these criteria.

Participants also had comprehensive neuropsychological assessments across multiple cognitive domains:

- Verbal memory: delayed recall in the Chinese Version Verbal Learning Test [3].
- Language: Boston Naming Test, and category (animal) Verbal Fluency Test.
- Visuospatial function: Taylor Complex Figure Test.
- Executive function: Backward Digit and Clock Drawing Test

Cognitive impairment in each domain was defined as a score in each test below 1.5 standard deviations of age- an

REFERENCES

1 American Psychiatric Association. Diagnostic and statistical manual of mental disorders. 3rd ed. Revised DSM-III-R. Washington DC: American Psychiatric Press, 1987.

2. Liu HC, Lin KN, Teng EL, Wang SJ, Fuh JL, Guo NW, Chou P, Hu HH, Chiang BN. Prevalence and subtypes of dementia in Taiwan: a community survey of 5297 individuals. J Am Geriatr Soc. 1995; 43:144–9. https://doi.org/10.1111/j.1532-5415.1995.tb06379.x. PMID:7836638

3. Chang CC, Kramer JH, Lin KN, Chang WN, Wang YL, Huang CW, Lin YT, Chen C, Wang PN. Validating the Chinese version of the Verbal Learning Test for screening Alzheimer's disease. J Int Neuropsychol Soc. 2010; 16:244–51. https://doi.org/10.1017/S1355617709991184 PMID:20003579
